# Recent Approaches for Chemical Speciation and Analysis by Electrospray Ionization (ESI) Mass Spectrometry

**DOI:** 10.3389/fchem.2020.625945

**Published:** 2021-01-20

**Authors:** Serena Indelicato, David Bongiorno, Leopoldo Ceraulo

**Affiliations:** Dipartimento di Scienze e Tecnologie Biologiche Chimiche e Farmaceutiche (STEBICEF), Università degli studi di Palermo, Palermo, Italy

**Keywords:** electrospray ionization, metals speciation, metallomics, mass spectrometry, speciation analysis

## Abstract

In recent years, the chemical speciation of several species has been increasingly monitored and investigated, employing electrospray ionization mass spectrometry (ESI-MS). This soft ionization technique gently desolvates weak metal–ligand complexes, taking them in the high vacuum sectors of mass spectrometric instrumentation. It is, thus, possible to collect information on their structure, energetics, and fragmentation pathways. For this reason, this technique is frequently chosen in a synergistic approach to investigate competitive ligand exchange-adsorption otherwise analyzed by cathodic stripping voltammetry (CLE-ACSV). ESI-MS analyses require a careful experimental design as measurement may face instrumental artifacts such as ESI adduct formation, fragmentation, and sometimes reduction reactions. Furthermore, ESI source differences of ionization efficiencies among the detected species can be misleading. In this mini-review are collected and critically reported the most recent approaches adopted to mitigate or eliminate these limitations and to show the potential of this analytical technique.

## Introduction

The IUPAC has defined the term “speciation analysis” as the “analytical activities of identifying and/or measuring the quantities of one or more individual chemical species in a sample.” It is also defined as “speciation of an element” the “distribution of an element amongst defined chemical species in a system.” Taking into account the development of the field and the wave of other -omic sciences, the term “metallomics” has been recently coined, defining “metallome” as the ensemble of metals and metalloids present in cells or tissues taking into consideration their nature, quantity, and localization.

To accomplish the complexity of this new research field, several new analytical methods have been developed, and integrated mass spectrometric tools were found to be fitting for this purpose.

In particular, for metallomics approaches, the combined use of chromatographic (or electrophoresis) separation and inductively coupled plasma-mass spectrometry (ICP-MS) is useful, whereas electrospray ionization-mass spectrometry (ESI-MS) allows the discrimination of species containing the same metal and to obtain structural elucidation.

## Electrospray Ionization

ESI has been developed as a soft ionization technique (Whitehouse et al., 1985) that gently takes into the gas phase metal–ligand complexes and allows gathering a wealth of information on their dissociation energetics, shapes, and fragmentation pathways. ESI-MS analyses require a careful experimental design as instrumental artifacts, such as adduct formation, source fragmentation, and sometimes reduction reactions, can occur.

Attention also must be paid to quantitative determination as differences in ionization efficiencies among the detected species can lead to misleading results. We here report the most recent approaches adopted to mitigate or eliminate these drawbacks. The potential of complementing ESI-MS results with quantum mechanical information and the coupling of the ESI sources with ion mobility (IM), high-resolution mass spectrometry (HR-MS), or tandem mass spectrometry (MS-MS) experiments, are also evidenced to provide unique information on the gas phase complexes.

The coupling of ICP and ESI sources with MS analyzers allows collapsing each ion into a single signal with a specific m/z value and precise intensity. This is immensely helpful to address the complex speciation problem associated with multiple complexation reactions that can take place in a solution. ESI as a “soft” ionization technique provides valuable information concerning the extracting ligands or complex stoichiometry, and ICP-MS analysis can give information only on the presence of the metal and on its abundance. However, one of the most debated arguments concerning ESI-MS spectra is the effective correspondence between the ionic species therein evidenced and the status of the correspondent ions or molecules in the bulk solution (Bongiorno et al., 2011a). Di Marco and Bombi (2006) have evidenced that perturbations of solution composition with respect to equilibrium take place during the ionization process. It is indeed common in the application of ESI-MS to ascertain differences between the relative abundance of the signals recorded in the spectra and the actual relative concentration of the species present in the condensed phase. These quantitative differences are due to differing gas-phase acidities/basicities, cation/anion affinities of the ionizing species, that lead to differing ionization efficiencies of the investigated species (Oss et al., 2010). Besides this, even large qualitative differences between solution phase and gas phase have been observed, self-assembly of alkali salts (Anacleto et al., 1992) or surfactant molecules being some of the most notable ones (Borysik and Robinson, 2012, Bongiorno et al., 2016). For these reasons, ESI requires a careful setup of experimental conditions to obtain reliable results. One of the most important parameters to optimize is the cone voltage that defines the so-called “*soft and hard*” ESI conditions (Bongiorno et al., 2011b). This potential is applied between the orifice and the skimmers. It can be useful, increasing ions' internal energy, to reduce the presence of residual clusters but can also lead to a more effective ion fragmentation and, therefore, to marked differences between abundances in solution and the gas phase (Indelicato et al., 2016). It follows that, despite the soft nature of ESI, fragmentation and/or polymerization phenomena may occur, and the spectra of species, that are sensitive to different instrumental parameters, may have different response factors (Espinosa et al., 2016). For this reason, a careful evaluation of the cone voltage has been crucial to determine polychalcogenids in solutions and to get reliable information for polysulfide ion speciation (Gun et al., 2004; Dorhout et al., 2017). Other authors (Wen et al., 2019) lowered cone voltages and temperatures to preserve the solution state at maximum.

The nature of solvents, cosolvents, and pH must be carefully evaluated as they are strongly related to ESI ionization efficiency. The introduction of methanol as a cosolvent is known to alter the solvent structure of water, leading to changes in both complexation kinetics and thermodynamics (Hawlicka and Swiatla-Wojcik, 2002; Accorsi et al., 2005; Wang et al., 2014). The pH variation directs the formation of protonated species and can have a strong influence on the relative abundance of formed complexes, leaving qualitatively unmodified the observed species (Espinosa et al., 2016). Besides this, the flux can have a small influence on the relative abundances of the aggregates (Bongiorno et al., 2005). Once these experimental factors are carefully defined, ESI-MS provides a reliable tool to extract quantitative information.

## Coupling ESI With Mass Spectrometry Analyzers

**Scheme 1:**
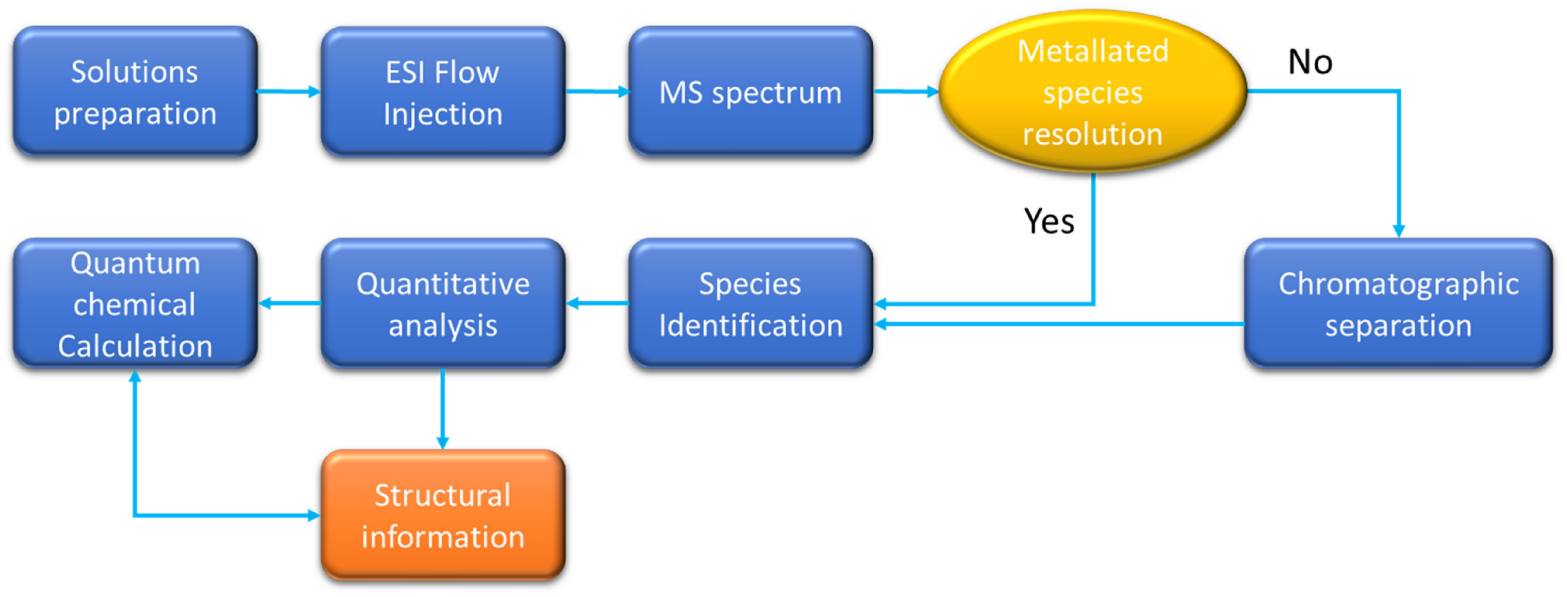
ESI-MS workflow for metal speciation.

The general approach followed by most of the authors developing ESI-MS methods to identify and characterize metallated species is represented in Scheme 1.

ESI sources have been coupled to several types of MS analyzers, and therefore, metal speciation experiments have been conducted in low-resolution MS, tandem MS (MS-MS), or high-resolution MS. However, there are some limitations for low-resolution MS for exploratory speciation analysis as evidenced by Bierla et al. (2018). Most of the drawbacks are due to slow scanning speed during HPLC runs, a blurred isotopic pattern due to background from concomitant species, and low sensitivity in full scan mode. It is possible to overcome these limitations by adopting multiple low-resolution analyzer systems (for MS-MS experiments) or adopting instruments with an increasing resolution power, such as time of flight (TOF), Fourier transform-orbital traps (FT-MS), and Fourier transform-ion cyclotron resonance (FT-ICR) mass spectrometers (de Hoffman and Stroobant, 2007). These different technological approaches lead to differing results in terms of resolution. Modern TOF instruments take advantage of a reflection grid to refocus ions in the space with the same mass, leading to a final resolution power of up to 50,000 full width at half maximum (FWHM). FT-MS traps ions in an orbital trap (generating a spindle-shaped electrostatic field). Ion masses are determined by applying the Fourier transform to the complex waveform of the image current, generated on the surface of the outer electrode by the ions orbiting in the trap. This approach leads to resolution of up to 1,000,000 FWHM. FT-ICR instrumentation takes the resolution a step further, up to and over 5,000,000 FWHM, but requires superconducting high field magnets to trap ions while Fourier transformation is applied to the waveform generated as image potential by the ions orbiting altogether in the magnetic field, each one with its own natural ion cyclotron resonance frequency.

## ESI-MS-MS for Metal Speciation

In several cases, the MS-MS approach for metal speciation is sufficient, guarantees exceptional sensitivity, and is well-suited for quantitative analysis. At least two independent approaches have been described by Liu et al. (2018) for speciation determination and quantitation of arsenic and its metabolites employing MS-MS.

Tsednee et al. (2016) developed an analytical application for identifying several transition metal (Co, Cu, Fe, Ni, Zn) complexes with deoxymugineic acid or nicotinamide by tandem mass spectrometry (ESI-MS-MS). It monitored, by multireaction monitoring (MRM), the release of free metals from the corresponding metal–ligand complexes. This MS-MS method allowed easily separating metal species whose mass spectra peaks were clustered together.

Tie et al. (2015) shows that HPLC-ESI-MS-MS is a sensitive and accurate method for the identification and quantification of the speciation of selenium. They monitored Se-methyselenocysteine (Se-MeSeCys) and selenomethionine (Se-Met) in soybean proteolytic digests through MRM mode. The evaluation of the fragmentation pattern of precursor ions (m/z 184 for Se-MeSeCys and m/z 198 for Se-Met) led to the selection of fragments due to the neutral loss of ammonia. Therefore, the transitions at m/z values of 184→167 for Se-MeSeCys and m/z values of 198→181 for Se-Met were monitored.

Quantitation of the appropriate HPLC peaks shows that inorganic selenium absorbed by the soybean has been biotransformed mainly into Se-MeSeCys. This species represented 66.4% of the selenium in Se-protein and 29.2% of the total selenium in the soybean.

## ESI Coupled to High-Resolution Mass Spectrometry Analyzers

Although exploiting MS-MS sensitivity is still an actual approach, in metal speciation, it is far more common to take advantage of high-resolution sectors, which are capable of well resolving isotopic clusters even in multiply charged adduct peaks.

The most common application of the simplest high-resolution mass spectrometry technology is the development of screening methods. Several authors followed this route. Raymond et al. (2018) developed a screening method for the characterization of beryllium complexes with aminopolycarboxylate and some related ligands. The approach requires only tiny amounts of material in analyte solutions and provides a quick and safe strategy for screening beryllium complexes. With a similar setup, Jo et al. (2019) investigated metal speciation of palladium in Pd-catalyzed pharmaceutical processes to verify the removal of elemental impurities from the reaction product mixture. They used metal speciation data to provide both critical information on the fate of each elemental impurity and a deeper understanding of the catalytic mechanism investigated. Using an ESI-TOF device, Wen et al. (2019) semi-quantitatively detected more than 30 types of aqueous vanadium species with <5% relative error. This led to a straightforward unambiguous molecular formula and ionic composition determination. Indelicato et al. (2014) investigated by ESI-MS, tandem mass spectrometry (ESI-MS-MS), and energy-resolved mass spectrometry (ER-MS) some lanthanide-functionalized surfactants: the ytterbium and erbium salts of bis(2-ethylhexyl)-sulfosuccinate (AOT). Evaluating the cone voltage effect on the metallated surfactant aggregation, they obtained detailed information on the stability and structural features of positively and negatively singly charged metallated species evidencing the formation of very large aggregates containing up to 5 Yb^3+^ or Er^3+^ ions.

Finally, Feng et al. (2015) exploited a similar instrumental setup to identify Al species in a complex mass spectrum. The authors introduce a novel theoretical calculation method based on the relative intensity of Gaussian-shaped peak clusters found in the spectra. Changes in m/z and molecular formulas of oligomers in five typical poly aluminum chloride (PAC) flocculants were easily deduced.

Exploiting more complex MS experiments and adopting time-resolved ESI-MS, Cao et al. (2016) monitored the “one-pot” method for the synthesis of polyoxometalates (POMs), produced using silicotungstates and vanadium salts. These authors discovered that the reaction conditions, such as concentration, temperature, and reaction time, sensitively changed the speciation.

The latest development of TOF technology, the so-called ion mobility mass spectrometry (IMMS) allows correlating the time of flight (drift time) of the ions within a “high pressure” mobility sector to determine collisional cross-sections of several type of ions ranging from peptides, small and large clusters, up to protein complexes (Lapthorn et al., 2013, Bongiorno et al., 2014).

Davis and Clowers (2018) recently used this cutting-edge approach for the rapid speciation of uranyl complexes. The authors were capable of stabilizing simple uranyl complexes during the ionization process and ion-mobility separation to aid speciation and isotope profile analysis. They measured mobilities of different uranyl species in simple mixtures by promoting stable gas-phase conformations with the addition of sulfoxides [i.e., dimethyl sulfoxide (DMSO), dibutyl sulfoxide (DBSO), and methyl phenyl sulfoxide (MPSO)]. As an outcome, this setup allowed the determination of the reduced mobilities of uranyl salts.

Opposite to the fast sensitivity-oriented approach of quadrupole ESI-MS or ESI-(q)TOF experiments, the adoption of FT-MS high-resolution analyzers allows for the development of more complex gas-phase experiments, opening a wide range of investigations allowed by the trapping of the ions in the analyzer for times that arrive to seconds. Waska et al. (2016) exploited high-resolution FT-ICR to overcome ESI-MS artifact and to characterize the equilibria of the model ligand citrate, EDTA, 1-nitroso-2-naphthol, and salicylaldoxime with iron (Fe^3+^) and copper (Cu^2+^). This approach allowed the detection of the whole metal–organic compounds. A cosolvent effect was ascertained, and methanol-containing samples gave higher sensitivities compared to those containing only water. It is important, however, to underline that, in comparing conditional stability constants determined by competitive ligand exchange-adsorptive cathodic stripping voltammetry (CLE-ACSV) with that of FT-ICR-MS determination, a difference was found. Therefore, the FT-ICR-MS-derived conditional stability constants can only be compared between similarly processed sample types.

Mapolelo et al. (2009) exploited the high-resolution capabilities of a custom-built FT-ICR analyzer, coupled with an infrared multiphoton dissociation CO_2_ continuous wave laser to gather the most information on the interaction of naphthenic acids with divalent (Ca^2+^, Fe^2+^, Mg^2+^) or monovalent (Na^+^, K^+^) ions in produced waters. These authors evidenced calcium naphthenate deposits that consist mainly of a C80 tetraprotic acid known as ARN acid bound to Ca^2+^. It was also possible to identify low-molecular-weight ARN acids with a C60-77 hydrocarbon skeleton in one calcium naphthenate deposit.

## ESI-MS and Quantum Mechanical Calculations

As it is evidenced so far, the coupling of ESI-MS information with data obtained from synergistic techniques, such as ICP-MS, NMR, X-RAY, and CLE-ACSV, is common practice. It is not a surprise to find out that the information obtained from ESI-MS speciation experiments is often compared to results of Ab-Initio or DFT quantum mechanical calculations. ESI-MS detects species in the gas phase, in which weak solvent interactions are absent.

This allows building quantum chemical simple and realistic models that are not impacted by the complex solvation. Theoretical calculations can be more easily compared to experimental results, and the model geometry suggests the structural information that is lacking in an ESI-MS spectrum. Exploiting these synergistic features, Raymond et al. (2019) investigated gas-phase coordination chemistry of Be^2+^ with 1,2- and 1,3-diketone ligands. Their results evidenced the tendency of beryllium to form stable polynuclear species with oxido, hydroxido, or diketonato ligands bridging the metal centers. In ESI-MS spectra were evidenced ions corresponding to predominant bis-chelated beryllium complexes known to be formed with the monoanionic 1,3-diketonate ligands.

ESI-MS measurements, along with differential functional theory calculations, have been exploited (Kumar et al., 2016) to understand the speciation of various uranyl species with α-hydroxyisobutyric acid. Quantum chemical calculations evidenced that uranyl complexes with 3 ligands (ML3 with M = UO2 and L = α-hydroxyisobutyric acid) are more energetically favorable over the ML2, which, in turn, are more favorable than ML1. The relative abundance of ML1 < ML2 < ML3 species in ESI-MS suggest a qualitative correlation between calculated free energies and observed complex relative stabilities. A similar approach was adopted to investigate the speciation of uranium–mandelic acid complexes (Kumar et al., 2017) determining structures and free energies of the complexes that were in fair agreement with the ESI spectra. Based on the energetics of this latter study, the authors further predicted the formation of T-shaped dimeric uranyl complexes in the complexation process.

## Desorption ESI and Ambient Mass Spectrometry Applications

To enhance ESI capabilities, some authors have developed some ancillary devices to couple with ESI sources. Jaklová Dytrtová et al. (2016) developed an electrochemical device that takes advantage of the high reactivity of electrochemically generated metallic ions *in statu nascendi*. This is suitable for ionization of various organic compounds (e.g., lipids, lipoproteins, pesticides, drugs, metabolites, lipids, lipoproteins) in biological and other matrices. The applicability of the electrochemical device is demonstrated by the electrochemical activation of pesticide cyproconazole (Cyp) in a soil solution matrix via formation and separation of its adducts with Ag and Cu cations without chromatographic support.

Finally, desorption electrospray ionization (DESI), an ESI-related technique that allows ionizing samples in the open environment and introducing them into the mass spectrometer reducing sample manipulation, is gaining momentum. Some authors studied Ru^+2^ complexes (Perry et al., 2011) evidencing that, in the short time scales of DESI, it is possible to detect trace levels (pmol) of short-lived intermediates characterized by lifetimes in the order of milliseconds. In a more recent work, Kazimi et al. (2019) exploited DESI to investigate, in the solid phase, a gold-based drug actually in clinical trials for its anticancer properties: auranofin. Auranofin was reacted with thiol-containing amino acids to evaluate the ligand exchange/scrambling reactions. These latter results evidence how the DESI-MS technique can be a game-changer in monitoring the reactions involving coordination compounds in the solid state.

## Conclusions

In conclusion, ESI-MS accompanied by its most recent variants, such as ambient MS (DESI), is proposing itself as a very informative method on metal complex–generated binding ligands, such as anions, bases, peptides, and proteins (see Table 1) The most important drawback of ESI-MS still lies in the possible difference between relative abundances of the species in the gas phase and in solution. This often requires validating the quantitative results with alternative spectroscopic techniques (Feng et al., 2015; Wen et al., 2019).

**Table 1:** Research articles summary, based on investigated metallic specie.

**Speciated metal**	**Analytical approach**
Ag	ESI-MS (Jaklová Dytrtová et al., 2016)
Al	ESI-TOF (Feng et al., 2015; Raymond et al., 2018)
As	ESI-MS-MS (Liu et al., 2018)
Au	DESI-MS (Kazimi et al., 2019)
Be	ESI-TOF (Raymond et al., 2018)
Ca	ESI-FT-ICR (Mapolelo et al., 2009)
Co	ESI-MS-MS (Tsednee et al., 2016)
Cu	ESI-MS (Jaklová Dytrtová et al., 2016), ESI-MS-MS (Tsednee et al., 2016), ESI-FT-ICR (Waska et al., 2016)
Er	ESI-TOF (Indelicato et al., 2014)
Fe	ESI-MS-MS, (Tsednee et al., 2016), ESI-FT-MS (Waska et al., 2016, Mapolelo et al., 2009)
K	ESI-FT-ICR (Mapolelo et al., 2009)
Mg	ESI-FT-ICR (Mapolelo et al., 2009)
Na	ESI-FT-ICR (Mapolelo et al., 2009)
Ni	ESI-MS-MS (Tsednee et al., 2016)
Pd	ESI-TOF (Jo et al., 2019)
Ru	DESI-MS (Perry et al., 2011)
Se	ESI-MS-MS (Tie et al., 2015)
U	ESI-TOF (Davis and Clowers, 2018)
V	ESI-TOF (Wen et al., 2019), ESI-TOF (time resolved) (Cao et al., 2016)
W	ESI-TOF (time resolved) (Cao et al., 2016)
Yb	ESI-TOF (Indelicato et al., 2013)
Zn	ESI-MS-MS (Tsednee et al., 2016)

ESI is especially informative when matrix or ion suppression effects are tolerable or negligible. When the matrix proves to be a serious drawback in the ESI determination of the speciated metals, the complementary information obtained by ICP-MS is still fundamental (Liu 2018). Some authors (Bierla et al., 2018) point out, however, that ESI-MS starts outpacing ICP-MS in terms of detection limits with the further advantage of the possibility to use the multiple reaction monitoring for quantification of adducts even in the case of incomplete separations. This increased sensitivity and the possibility of large-scale data acquisition is opening new opportunities even in tasks demanding high sensitivity, such as metallo-metabolomics and metallo-proteomics of body fluids and tissues of higher organisms (Bierla et al., 2018).

## Author Contributions

All the authors equally contributed to the bibliographical research of the references cited and to the article redaction.

## Conflict of Interest

The authors declare that the research was conducted in the absence of any commercial or financial relationships that could be construed as a potential conflict of interest.

## References

[B1] Accorsi A. Morrone B. Benzo M. Gandini C. Raffi G. B. Violante F. S. (2005). Simultaneous determination of unmodified sevoflurane and of its metabolite hexafluoroisopropanol in urine by headspace sorptive extraction-thermal desorption-capillary gas chromatography-mass spectrometry. J. Chromatogr. A 1071, 131–134. 10.1016/j.chroma.2004.09.039 15865184

[B2] Anacleto J. F. Pleasance S. Boyd R. K. (1992). Calibration of ion spray mass spectra using cluster ions. Org. Mass Spectrom. 27, 660–666. 10.1002/oms.1210270603

[B3] Bierla K. Godin S. Lobinski R. Szpunar J. (2018). Advances in electrospray mass spectrometry for the selenium speciation: focus on Se-rich yeast. Trends Anal. Chem. 104, 87–94. 10.1016/j.trac.2017.10.008

[B4] Bongiorno D. Ceraulo L. Giorgi G. Indelicato S. Ferrugia M. Ruggirello A.. (2011b). Effects of the net charge on abundance and stability of supramolecular surfactant aggregates in gas phase. J. Mass Spectrom. 46, 195–201. 10.1002/jms.1872 21259391

[B5] Bongiorno D. Ceraulo L. Giorgi G. Indelicato S. Turco Liveri V. (2011a). Do electrospray mass spectra of surfactants mirror their aggregation state in solution? J. Mass Spectrom. 46, 1262–1267. 10.1002/jms.2013 22223417

[B6] Bongiorno D. Ceraulo L. Indelicato S. Turco Liveri V. Indelicato S. (2016). Charged supramolecular assemblies of surfactant molecules in gas phase. Mass Spectrom. Rev. 35, 170–187. 10.1002/mas.21476 26113001

[B7] Bongiorno D. Ceraulo L. Ruggirello A. Liveri V. T. Basso E. Seraglia R.. (2005). Surfactant self-assembling in gas phase: electrospray ionization- and matrix-assisted laser desorption/ionization-mass spectrometry of singly charged AOT clusters. J. Mass Spectrom. 40, 1618–1625. 10.1002/jms.965 16320296

[B8] Bongiorno D. Indelicato S. Giorgi G. Scarpella S. Liveri V. T. Ceraulo L. (2014). Electrospray ion mobility mass spectrometry of positively charged sodium bis(2-ethylhexyl)sulfosuccinate aggregates. Eur. J. Mass Spectrom. (Chichester) 20, 169–175. 10.1255/ejms.1261 24895777

[B9] Borysik A. J. Robinson C. V. (2012). Formation and dissociation processes of gas-phase detergent micelles. Langmuir 28, 7160–7167. 10.1021/la3002866 22512598

[B10] Cao J. Liu C. Jia Q. Di (2016). Complex solution chemistry behind the simple “one-pot” synthesis of vanadium-substituted polyoxometalates unraveled by electrospray ionization mass spectrometry. Rapid Commun. Mass Spectrom. 30, 14–19. 10.1002/rcm.7641 27539408

[B11] Davis A. L. Clowers B. H. (2018). Stabilization of gas-phase uranyl complexes enables rapid speciation using electrospray ionization and ion mobility-mass spectrometry. Talanta 176, 140–150. 10.1016/j.talanta.2017.07.090 28917733

[B12] de Hoffman E. Stroobant V. (2007). Mass Spectroscopy: Principles and Applications, 3rd Edn. NewYork, NY: John Wiley & Sons Ltd.

[B13] Di Marco V. B. Bombi G. G. (2006). Electrospray mass spectrometry (ESI-MS) in the study of metal–ligand solution equilibria. Mass Spectrom. Rev. 25, 347–379. 10.1002/mas.20070 16369936

[B14] Dorhout P. K. Ford N. B. Raymond C. C. (2017). Understanding the polychalcogenides as building blocks to solid state materials: speciation of polychalcogenides in solutions. Coord. Chem. Rev. 352, 537–550. 10.1016/j.ccr.2017.10.017

[B15] Espinosa M. S. Servant R. Babay P. A. (2016). Study of metal-ligand species by ESI-MS: the case of La, Nd, and Th complexes with EDTA. Microchem. J. 129, 151–157. 10.1016/j.microc.2016.06.018

[B16] Feng C. Bi Z. Tang H. (2015). Electrospray ionization time-of-flight mass spectrum analysis method of polyaluminum chloride flocculants. Environ. Sci. Technol. 49, 474–480. 10.1021/es503681p 25436867

[B17] Gun J. Modestov A. D. Kamyshny A. Ryzkov D. Gitis V. Goifman A. (2004). Electrospray ionization mass spectrometric analysis of aqueous polysulfide solutions. Microchim. Acta 146, 229–237. 10.1007/s00604-004-0179-5

[B18] Hawlicka E. Swiatla-Wojcik D. (2002). MD Simulation studies of selective solvation in methanol–water mixtures: an effect of the charge density of a solute. J. Phys. Chem. A 106, 1336–1345. 10.1021/jp012662w

[B19] Indelicato S. Bongiorno D. Ceraulo L. Calabrese V. Piazzese D. Napoli A. (2016). Electrospray ion mobility mass spectrometry of positively and negatively charged (1R,2S)-dodecyl(2-hydroxy-1-methyl-2-phenylethyl)dimethylammonium bromide aggregates. Rapid Commun. Mass Spectrom. 30, 230–238. 10.1002/rcm.7422 26661990

[B20] Indelicato S. Bongiorno D. Indelicato S. Drahos L. Turco Liveri V. Turiák L.. (2013). Degrees of freedom effect on fragmentation in tandem mass spectrometry of singly charged supramolecular aggregates of sodium sulfonates. J. Mass Spectrom. 48, 379–383. 10.1002/jms.3161 23494795

[B21] Indelicato S. Bongiorno D. Liveri V. T. Mele A. Panzeri W. Castiglione F.. (2014). Self-assembly and intra-cluster reactions of erbium and ytterbium bis(2-ethylhexyl)sulfosuccinates in the gas phase. Rapid Commun. Mass Spectrom. 28, 2523–2530. 10.1002/rcm.7045 25366399

[B22] Jaklová Dytrtová J. Jakl M. Navrátil T. Cvačka J. Pačes O. (2016). An electrochemical device generating metal ion adducts of organic compounds for electrospray mass spectrometry. Electrochim. Acta 211, 787–793. 10.1016/j.electacta.2016.06.108

[B23] Jo J. Tu Q. Xiang R. Li G. Zou L. Maloney K. M. (2019). Metal Speciation in Pharmaceutical Process Development: Case Studies and Process/Analytical Challenges for a Palladium-Catalyzed Cross-Coupling Reaction. Organometallics 38, 185–193. 10.1021/acs.organomet.8b00638

[B24] Kazimi S. G. T. Iqbal M. S. Mulligan C. C. Frank Shaw C. Iram F. Stelmack A. R.. (2019). Ligand exchange/scrambling study of gold(I)-phosphine complexes in the solid phase by DESI-MS analysis. J. Am. Soc. Mass Spectrom. 30, 2289–2296. 10.1007/s13361-019-02319-y 31502222

[B25] Kumar P. Jaison P. G. Telmore V. M. Alamelu D. Aggarwal S. K. Sadhu B. (2016). Gas phase reactions of uranyl with α-hydroxyisobutyric acid using electrospray ionization mass spectrometry and density functional theory. J. Radioanal. Nucl. Chem. 308, 303–310. 10.1007/s10967-015-4664-6

[B26] Kumar P. Jaison P. G. Telmore V. M. Sadhu B. Sundararajan M. (2017). Speciation of uranium-mandelic acid complexes using electrospray ionization mass spectrometry and density functional theory. Rapid Commun. Mass Spectrom. 31, 561–571. 10.1002/rcm.7817 28035726

[B27] Lapthorn C. Pullen F. Chowdhry B. Z. (2013). Ion mobility spectrometry-mass spectrometry (IMS-MS) of small molecules: separating and assigning structures to ions. Mass Spectrom. Rev. 32, 43–71. 10.1002/mas.21349 22941854

[B28] Liu Q. Lu X. Peng H. Popowich A. Tao J. Uppal J. S. (2018). Speciation of arsenic—a review of phenylarsenicals and related arsenic metabolites. Trends Anal. Chem. 104, 171–182. 10.1016/j.trac.2017.10.006

[B29] Mapolelo M. M. Stanford L. A. Rodgers R. P. Yen A. T. Debord J. D. Asomaning S. (2009). Chemical speciation of calcium and sodium naphthenate deposits by electrospray ionization FT-ICR mass spectrometry. Energy Fuels 23, 349–355. 10.1021/ef800642b

[B30] Oss M. Kruve A. Herodes K. Leito I. (2010). Electrospray ionization efficiency scale of organic compounds. Anal. Chem. 82, 2865–2872. 10.1021/ac902856t 20218595

[B31] Perry R. H. Splendore M. Chien A. Davis N. K. Zare R. N. (2011). Detecting reaction intermediates in liquids on the millisecond time scale using desorption electrospray ionization. Angew. Chem. 123, 264–268. 10.1002/ange.201004861 21110361

[B32] Raymond O. Brothers P. J. Buchner M. R. Lane J. R. Müller M. Spang N.. (2019). Electrospray ionization mass spectrometric study of the gas-phase coordination chemistry of Be 2+ ions with 1,2- and 1,3-diketone ligands. Inorg. Chem. 58, 6388–6398. 10.1021/acs.inorgchem.9b00578 30963770

[B33] Raymond O. Henderson W. Brothers P. J. Plieger P. G. (2018). Electrospray Ionisation Mass Spectrometric (ESI MS) screening and characterisation of beryllium complexes with potentially encapsulating aminopolycarboxylate and related ligands. Eur. J. Inorg. Chem. 2018, 1120–1130. 10.1002/ejic.201701435

[B34] Tie M. Li B. Zhuang X. Han J. Liu L. Hu Y. (2015). Selenium speciation in soybean by high performance liquid chromatography coupled to electrospray ionization-tandem mass spectrometry (HPLC-ESI-MS/MS). Microchem. J. 123, 70–75. 10.1016/j.microc.2015.05.017

[B35] Tsednee M. Huang Y. C. Chen Y. R. Yeh K. C. (2016). Identification of metal species by ESI-MS/MS through release of free metals from the corresponding metal-ligand complexes. Sci. Rep. 6, 1–13. 10.1038/srep26785 27240899 PMC4886218

[B36] Wang C.-H. Bai P. Siepmann J. I. Clark A. E. (2014). Deconstructing hydrogen-bond networks in confined nanoporous materials: implications for alcohol–water separation. J. Phys. Chem. C 118, 19723–19732. 10.1021/jp502867v

[B37] Waska H. Koschinsky A. Dittmar T. (2016). Fe- and Cu-complex formation with artificial ligands investigated by ultra-high resolution fourier-transform ion cyclotron resonance mass spectrometry (FT-ICR-MS): implications for natural metal-organic complex studies. Front. Mar. Sci. 3, 1–19. 10.3389/fmars.2016.00119

[B38] Wen J. Ning P. Cao H. Zhao H. Sun Z. Zhang Y. (2019). Novel method for characterization of aqueous vanadium species: a perspective for the transition metal chemical speciation studies. J. Hazard. Mater. 364, 91–99. 10.1016/j.jhazmat.2018.09.069 30342292

[B39] Whitehouse C. M. Dreyer R. N. Yamashita M. Fenn J. B. (1985). Electrospray interface for liquid chromatographs and mass spectrometers. Anal. Chem. 57, 675–679. 10.1021/ac00280a023 2581476

